# Evaluation of six candidate DNA barcode loci for identification of five important invasive grasses in eastern Australia

**DOI:** 10.1371/journal.pone.0175338

**Published:** 2017-04-11

**Authors:** Aisuo Wang, David Gopurenko, Hanwen Wu, Brendan Lepschi

**Affiliations:** 1 Wagga Wagga Agricultural Institute, NSW Department of Primary Industries, Wagga Wagga, Australia; 2 Graham Centre for Agricultural Innovation (An alliance between NSW Department of Primary Industries and Charles Sturt University), Wagga Wagga, New South Wales, Australia; 3 Australian National Herbarium, Centre for Australian National Biodiversity Research, Canberra, Australian Capital Territory, Australia; Chinese Academy of Medical Sciences and Peking Union Medical College, CHINA

## Abstract

Invasive grass weeds reduce farm productivity, threaten biodiversity, and increase weed control costs. Identification of invasive grasses from native grasses has generally relied on the morphological examination of grass floral material. DNA barcoding may provide an alternative means to identify co-occurring native and invasive grasses, particularly during early growth stages when floral characters are unavailable for analysis. However, there are no universal loci available for grass barcoding. We herein evaluated the utility of six candidate loci (*atpF* intron, *matK*, *ndhK-ndhC*, *psbE—petL*, ETS and ITS) for barcode identification of several economically important invasive grass species frequently found among native grasses in eastern Australia. We evaluated these loci in 66 specimens representing five invasive grass species (*Chloris gayana*, *Eragrostis curvula*, *Hyparrhenia hirta*, *Nassella neesiana*, *Nassella trichotoma*) and seven native grass species. Our results indicated that, while no single locus can be universally used as a DNA barcode for distinguishing the grass species examined in this study, two plastid loci (*atpF* and *matK*) showed good distinguishing power to separate most of the taxa examined, and could be used as a dual locus to distinguish several of the invasive from the native species. Low PCR success rates were evidenced among two nuclear loci (ETS and ITS), and few species were amplified at these loci, however ETS was able to genetically distinguish the two important invasive *Nassella* species. Multiple loci analyses also suggested that ETS played a crucial role in allowing identification of the two *Nassella* species in the multiple loci combinations.

## Introduction

A variety of invasive grasses in Eastern Australia, including *Nassella trichotoma* (Nees) Hack. ex Arechav. (Serrated tussock), *Nassella neesiana* (Trin. & Rupr.) Barkworth (Chilean Needle Grass), *Hyparrhenia hirta* (L.) Stapf (Coolatai grass), *Eragrostis curvula* (Schrad.) Nees (African lovegrass), *Chloris gayana* Kunth (Rhodes grass) and others have caused serious damage to the environment and agricultural industry [1]. Both *N*. *trichotoma* and *N*. *neesiana* are native to South American countries (Argentina, Uruguay, Peru etc.), and have become naturalised weeds in Australia (particularly in eastern states such as New South Wales, Australian Capital Territory, Victoria and Tasmania)[2, 3]. They also occur as weeds in New Zealand, South Africa, USA and several European countries [4]. As these two weeds are capable of displacing palatable grasses from pastures, decreasing productivity of grazing livestock, and significantly degrading biodiversity in native grasslands, they have been listed as WONS (Weeds of National Significance in Australia)[4] requiring nationally coordinated control efforts. *E*. *curvula* is native to South Africa. As an invasive weed in Australia, New Zealand, and some parts of USA, it competes with native pasture species, reduces livestock productivity and posts fire hazard to environment [5]. Being a native in tropical and temperate Africa and the Mediterranean region, *H*. *hirta* has become naturalized in Australia, Mexico, the Caribbean and parts of South America [6]. It poses a major threat to natural biodiversity in stock routes, nature reserves and National Parks due to its tolerance to drought, fire and herbicide. Originated from Africa, *C*. *gayana* is a widely naturalised weed infesting pastures and native vegetation zones in Australia, New Zealand, USA, and several Pacific islands (Weeds of Australia, https://keyserver.lucidcentral.org/weeds/).

Identification of invasive and native grasses is crucial in weed management as misidentification of weeds may delay the control of invasive weeds while causing unnecessary eradication of native grasses with similar morphology[1]. Unfortunately, the five invasive grasses can be easily misidentified to a variety of native grasses in Australia. For examples, *N*. *trichotoma* and *N*. *neesiana* are both very similar in appearance to *Austrostipa* spp and in some cases similar to, *Poa* and *Eragrostis* species; *E*. *curvula* is alike to other tussock-like grasses; *H*. *hirta* is comparable to kangaroo grass (*Themeda australis*) and *C*. *gayana* is similar to feathertop Rhodes grass (*Chloris virgata*) (NSW WeedWise, http://weeds.dpi.nsw.gov.au/).

Weed abatement officers in eastern Australia and elsewhere generally identify grasses based mainly on visual examination of diagnostic floral characters present in mature specimens. As a result, grasses at earlier stages of development may not be readily identifiable, and in those instances, invasive grass infestations can go undetected during weed abatement surveys. DNA barcoding [7] provides a potential alternative method for distinguishing invasive grasses from native grasses without the dependency of suitable growth-stage samples. As a genetic based method, DNA barcoding can accurately and rapidly identify samples to species, even from trace amounts or degraded sample tissue [8, 9]. The success of DNA barcoding relies on the amplification of specific barcoding locus or loci in the genomes of the target species. The mitochondrial locus, *COI* (Cytochrome *c* Oxidase subunit I), has been successfully applied to provide species-level resolution in many animal species [10, 11]. In land plants, however, *COI* has proved to be less effective due to conservative rates of nucleotide substitution in land plant mitochondrial DNA [12].

The lack of universal barcoding locus in plants impelled the search for alternative DNA barcoding regions outside the mitochondrial genome [13–15]. As a result, the Consortium for the Barcode of Life (CBOL) recommended use of plastid coding genes maturase K (*matK*) and ribulose-1,5-bisphosphate carboxylase oxygenase (*rbcL*) as core plant barcodes[14] and the nuclear internal transcribed spacers 1–2 (ITS)[14] as supplementary loci. Nevertheless, finding more robust loci to increase the resolution in distinguishing particular groups of plant species (particularly higher plants) is still an ongoing process [16].

Syme, *et al*. (2013) [4] assessed the accuracy of standard barcoding loci (*rbcL*, *matK* and ITS) in a study aiming to test the efficiency of three sequence matching algorithms (BLAST, Neighbour Joining and Bayesian Likelihood) for genetic identification of stipoid grasses. They reported the best DNA barcode accuracy using ITS, partial accuracy using *matK*, and least accuracy using *rbcL*. Wang, *et al*. (2014) [1] screened 18 loci for the possibility of using DNA barcoding technology to identify invasive weeds and native grasses (up to 29 grass species) collected from eastern Australia. Based on PCR reliability and polymorphism levels, they advocated the use of *matK* and two other cpDNA loci [*ndhK-ndhC* intergenic spacer (referred as *ndhK*) and *psbE*—*petL* intergenic spacer (referred to here as as *psbE*)] as preferred grass DNA barcode targets over *rbcL* and ITS.

Here, we evaluated the chloroplast and nuclear loci (ITS, *matK*, *ndhk* and *psbE*), which were recommended by previous studies, on five major invasive grasses (*N*. *trichotoma*, *N*. *neesiana*, *E*. *curvula*, *H*. *hirtai*, *C*. *gayana*) and several co-occurring native grasses. We also tested two new loci, *atpF* intron (referred as *atpF*) and external transcribed spacer (ETS), which were reported to be effective in the genetic diversity study of *Lolium perenne* and other related grass species [17], and the phylogenetic studies of *Poa* [18]. We hope the availability of robust DNA barcoding loci will help weeds abatement officers to identify the five invasive weeds at early growth stages, which is important to Australian biosecurity (including border entry points for quarantine purposes) and weed control agencies as early detection in bio-surveillance for weed control will ensure an early intervention by control agencies.

## Materials and methods

### Sample collection

A total of 66 specimens representing five invasive grass species in Eastern Australia (*Chloris gayana*, *Eragrostis curvula*, *Hyparrhenia hirta*, *Nassella neesiana*, *Nassella trichotoma*) and seven native grass species (*Austrostipa densiflora*, *Anthosachne scabra*, *Microlaena stipoides*, *Poa sieberiana*, *Rytidosperma caespitosum*, *Rytidosperma pallidum* and *Themeda triandra*) were collected from the Australian Capital Territory, New South Wales, Queensland and Victoria in eastern Australia under the permit issued by Department of Primary Industry (Permit No. INT14/8307) (see S1 Table for collection information and GenBank accession numbers). The seven native grasses were selected for examination here, as each is morphologically similar to one or more of the invasive grasses and potentially affected by their presence in areas of Eastern Australia where they overlap. These samples included field sampled specimens (N = 62) and vouchered herbarium specimens from the Australian National Herbarium (N = 2) and the National Herbarium of Victoria (N = 2). Leaf samples (appropriately 0.3 cm^2^ in size) were preserved in > 70% EtOh and stored at the Wagga Wagga Agricultural Institute (NSW Department of Primary Industries) and allocated unique specimen identifiers (e.g. ww00001) for sample tracking. All specimen records and associated gene sequences have been submitted into the Barcode of Life Data systems (BOLD) [19].

### DNA extraction, target loci PCR, and sequencing

In preparation for DNA extractions, leaf tissue (< 1 mg) of each specimen was incubated (55°C) overnight in 280 μl of DXT tissue digest reagent (QIAGEN, Doncaster, Australia) with 1% added proteinase K (Sigma—Aldrich). Genomic DNA was isolated from specimen digestions using a Corbett Research 1820 X-tractor Gene robot and associated DX buffers (Qiagen, Doncaster, Australia).

Loci specific forward and reverse primers used in Polymerase Chain Reaction (PCR) amplification are listed in (Table 1) and were modified by addition of 17 bp forward (GTAAAACGACGGCCAGT) and reverse (CAGGAAACAGCTATGAC) vector M-13 5’ tails to simplify subsequent sequencing.

**Table 1:** Loci targeted for PCR using sourced primers. Forward (-F) and reverse (-R) primer directions indicated by suffix. Original primer sequences modified by addition of 17 bp vector M-13 5´ tails (tail sequences not shown here, refer to Materials and methods).

Locus	Primer name	Primer sequence 5'-3'	Source
*atpF* intron	TeaCpSSR27FP	AATGCCGAATCGACGACCTA	[17]
	TeaCpSSR27RP	CAATGGTCCCTCTACGCAAT	
*matK*	390-F	CGATCTATTCATTCAATATTTC	[20]
	1326-R	TCTAGCACACGAAAGTCGAAGT	
	3F-Kimf	CGTACAGTACTTTTGTGTTTACGAG	
	1R-Kimr	ACCCAGTCCATCTGGAAATCTTGGTTC	
*ndhK*-*ndhC*	TeaCpSSR29FP	GGTACCAATCCATAACGATC	[17]
	TeaCpSSR29RP	GCGCTAGTTTTTGTTGTTTT	
*psbE-petL*	TeaCpSSR31FP	GGTCGTGGAATGCTTTTCTT	
	TeaCpSSR31RP	TCCACGAATCTCAATGACCA	
ETS	Rets4-F	TTGGCTACGCGAGCGCATGAG	[21]
	18S-R	AGACAAGCATATGACTACTGGCAGG	[22]
ITS	26SE	TAGAATTCCCCGGTTCGCTCGCCGTTAC	[23]
	S3	AACCTGCGGAAGGATCATTG	[24]
	ITS 5a–F	TATCATTTAGAGGAAGGAG	[25]
	ITS 4—R	GCATATCAATAAGCGGAGGA	

PCR (15 μl aliquots) for each locus (except ITS) contained Invitrogen^™^ reagents (1 × PCR buffer 2.9 mM MgCI_2_, 0.2 mM dNTPs, 0.4 U of Platinum Taq polymerase), 0.1 μM each primer, and 2 μl of specimen DNA. PCR thermal cycling consisted of 2 min at 95°C, 40 x [94°C (30s), 50°C (30s), 72°C (1 min)] and 72°C (5 min). All PCRs were conducted using an Eppendorf Mastercycler EP Gradient S Thermal Cycler.

ITS PCR modifications [18] included the addition of 0.75 μl of DMSO (100%) (Sigma—Aldrich) and 1 μl of specimen DNA in 15 μl reactions, with thermal cycling set as 95°C (5 min), 35 × [94°C (30 s), 55°C (30 s), 72°C (1 min)], and 72°C (5 min).

PCR products stained with SYBR^®^ safe DNA Gel Stain (Invitrogen) were examined under UV light in a Bio—Rad Universal Hood II following electrophoresis through a 1.5% agarose gel, and in the presence of 1 kb size markers and negative controls. Successful PCR products were sequenced at the Brisbane, Queensland node of the Australian Genome Research Facility (AGRF).

### Data analysis

Forward and reverse sequence chromatograms at each locus were assembled and checked for signal quality using SeqMan (DNA STAR package, DNAStar Inc., Madison, WI, USA). At each locus, specimen consensus sequences were exported into BioEdit [26] for alignment using ClustalW [27] with default parameters. The aligned sequences were also manually edited in BioEdit to remove primer reads.

DNA barcode gap analysis was determined at each locus by plotting maximum intraspecific distance (*D*_intra_) against minimum nearest neighbour distance (*D*_NN_). Intra and inter-specific pairwise genetic distances used in barcode gap analyses were generated at BOLD and adjusted by the Kimura 2-parameter (K2P) model of nucleotide evolution.

**Fig 1:**
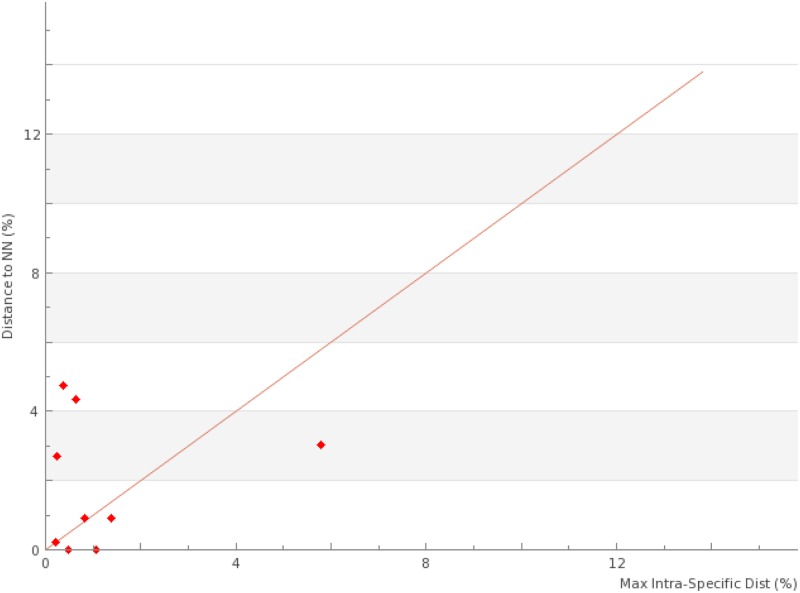
Maximum percent intraspecific distance (*D*_intra_), and percent distance to nearest genetic neighbour species for six loci across five invasive and seven native grass species. **A: *atpF*; B: *psbE*; C: *matK*; D:** ETS**; E: *ndhK*; F:** ITS.

At each locus, the nearest neighbour minimum interspecific *p–*distances (*D*_NN_) was plotted against maximum intraspecific *p–*distances (*D*_intra_) to determine presence or absence of a DNA barcode gap among species (Fig 1).

Species monophyly was tested at each locus using both Neighbor-joining (NJ) and Maximum Likelihood (ML) tree construction methods. K2P pairwise distances used in NJ tree constructions were computed in MEGA 6.0 [28]. ML trees were constructed using PhyML 3.1 [29], incorporating a GTR nucleotide substitution model (plus Gamma distribution). Bootstrap replication (N = 1000) was used to assess confidence of NJ clusters and ML clades.

The same phylogenetic analyses were also performed on multi locus combinations (di, tri, tetra and penta) of the six loci to determine if any locus combination outperformed single loci for resolving species monophyly.

## Results

### Sequence characteristics of the six loci

While only one primer set was tested for each of four loci (ETS, *atpF*, *ndhK* and *psbE*), two primer sets were applied to amplify the ITS and *matK* sequences across the 12 species (Table 1). PCR success rate varied across different loci and grass species (Table 2). *atpF* was the only locus that successfully amplified all tested grass species. High rates of PCR success were also apparent at *matK* and *ndhK*. ITS had the least PCR success, with only three of the twelve grass species (*C*. *gayana*, *E*. *curvula* and *N*. *trichotoma*) being successfully amplified at this locus (Table 2). The aligned sequence matrix of ETS was 491 nucleotides (nt) in length with 314 parsimony informative sites and 325 variable sites (Table 3), which represents the highest percentage value of the parsimony informative or variable nucleotide sites against the total length (66% and 68% respectively). The shortest alignment (356 nt) was at *ndhK*, which also had the least amount of parsimony informative and variable sites against the total length (11% and 15%) respectively. DNA barcode gap minimum interspecific p—distances [represented as nearest neighbor distance (D_NN_)] and maximum intraspecific p—distances (D_intra_) among the tested species across the six loci were presented in Table 4 and in Fig 1.

**Table 2:** PCR success rate of each locus against each species tested (Invasive grasses are indicated with *).

Species (specimen No.)	*atpF*	ETS	ITS	*matK*	*ndhK*	*psbE*
*Austrostipa densiflora (2)*	100%	100%	0%	100%	100%	0%
*Chloris gayana (3)	100%	67%	100%	0%	33%	0%
*Anthosachne scaber (4)*	75%	0%	0%	100%	25%	75%
*Eragrostis curvula (18)	39%	0%	100%	78%	89%	33%
*Hyparrhenia hirta (6)	100%	33%	0%	33%	83%	83%
*Microlaena stipoides (3)*	100%	0%	0%	100%	67%	0%
*Nassella neesiana (12)	100%	92%	0%	83%	92%	92%
*Nassella trichotoma (8)	100%	100%	13%	75%	88%	75%
*Poa sieberiana (2)*	100%	0%	0%	100%	0%	0%
*Rytidosperma caespitosum (2)*	100%	0%	0%	100%	0%	0%
*Rytidosperma pallidum (4)*	100%	0%	0%	100%	0%	0%
*Themeda triandra (2)*	100%	0%	0%	100%	0%	0%

**Table 3:** Information used to evaluate the utility of the six loci.

Items	ETS	ITS	*atpF*	*matK*	*ndhK*	*psbE*
No. of primers screened	1	2	1	2	1	1
Aligned length (bp)	491	687	457	838	356	687
Informative sites/variable sites	314/325	140/243	95/114	168/195	38/54	396/401
No. of Indels	70	48	82	6	10	74

**Table 4:** Percent distance of maximum percent intraspecific distance (*D*_intra_) and nearest genetic neighbour species (*D*_NN_) across five invasive and seven native grass species (N/A: not available for single specimen).

Locus	Species	*D*_intra_	Nearest species	*D*_NN_
***matK***	*Anthosachne scabra*	0.9	*Rytidosperma pallidum*	0.38
	*Austrostipa densiflora*	0.12	*Nassella trichotoma*	1.43
	*Eragrostis curvula*	1.28	*Hyparrhenia hirta*	4.49
	*Hyparrhenia hirta*	0.56	*Eragrostis curvula*	4.49
	*Microlaena stipoides*	1.28	*Austrostipa densiflora*	6.21
	*Nassella neesiana*	0.66	*Nassella trichotoma*	0.11
	*Nassella trichotoma*	0.97	*Nassella neesiana*	0.11
	*Poa sieberiana*	0.45	*Anthosachne scabra*	4.84
	*Rytidosperma caespitosum*	9.71	*Rytidosperma pallidum*	0
	*Rytidosperma pallidum*	9.45	*Rytidosperma caespitosum*	0
	*Themeda triandra*	6.47	*Rytidosperma pallidum*	0
**ETS**	*Austrostipa densiflora*	0.77	*Nassella neesiana*	7.09
	*Chloris gayana*	0	*Hyparrhenia hirta*	27.31
	*Hyparrhenia hirta*	0.7	*Chloris gayana*	27.31
	*Nassella neesiana*	0.21	*Nassella trichotoma*	2.92
	*Nassella trichotoma*	0.21	*Nassella neesiana*	2.92
**ITS**	*Chloris gayana*	0.87	*Eragrostis curvula*	16.74
	*Eragrostis curvula*	5.21	*Chloris gayana*	16.74
	*Nassella trichotoma*	N/A	*Eragrostis curvula*	20.7
***atpF***	*Anthosachne scabra*	0.25	*Austrostipa densiflora*	2.7
	*Austrostipa densiflora*	0.2	*Nassella trichotoma*	0.22
	*Chloris gayana*	0	*Eragrostis curvula*	3.03
	*Eragrostis curvula*	5.8	*Chloris gayana*	3.03
	*Hyparrhenia hirta*	0.38	*Eragrostis curvula*	4.73
	*Microlaena stipoides*	0.63	*Nassella neesiana*	4.34
	*Nassella neesiana*	1.05	*Nassella trichotoma*	0
	*Nassella trichotoma*	0.48	*Nassella neesiana*	0
	*Poa sieberiana*	0	*Themeda triandra*	2.45
	*Rytidosperma caespitosum*	0.83	*Rytidosperma pallidum*	0.9
	*Rytidosperma pallidum*	1.38	*Rytidosperma caespitosum*	0.9
	*Themeda triandra*	0	*Poa sieberiana*	2.45
***ndhK***	*Anthosachne scabra*	N/A	*Austrostipa densiflora*	2.13
	*Austrostipa densiflora*	0	*Nassella neesiana*	0.26
	*Chloris gayana*	N/A	*Eragrostis curvula*	2.53
	*Eragrostis curvula*	2.79	*Chloris gayana*	2.53
	*Hyparrhenia hirta*	0.75	*Eragrostis curvula*	3.2
	*Microlaena stipoides*	0.82	*Eragrostis curvula*	3.37
	*Nassella neesiana*	2.83	*Nassella trichotoma*	0
	*Nassella trichotoma*	4.85	*Nassella neesiana*	0
***psbE***	*Anthosachne scabra*	0.47	*Nassella neesiana*	42.41
	*Eragrostis curvula*	0.63	*Hyparrhenia hirta*	5.16
	*Hyparrhenia hirta*	1.16	*Eragrostis curvula*	5.16
	*Nassella neesiana*	2.07	*Nassella trichotoma*	0
	*Nassella trichotoma*	0.29	*Nassella neesiana*	0

ETS and ITS were the only loci to show evidence of a clear DNA barcode gap, as exemplified by the absence of overlap between *D*_intra_ and *D*_NN_. In contrast these two measures overlapped at each of the remaining four cpDNA loci, indicating instances where more variation was present within particular species than between their nearest genetic neighbor species.

Among the six loci, ETS showed good distinguishing power to separate all targeted invasive grasses (except for *E*. *curvula* where it failed to amplify) as clear barcode gaps separating maximum intraspecific and minimum interspecific distances were identified between the invasive species (Fig 1D). While the remaining loci differed in their abilities to separate different weeds species, they shared the same feature that they failed to distinguish two important invasive grasses *N*. *neesiana* and *N*. *trichotoma* (Fig 1A, 1B, 1C, 1E and 1F).

### Monophyly tests of species based on phylogenetic trees

**Fig 2:**
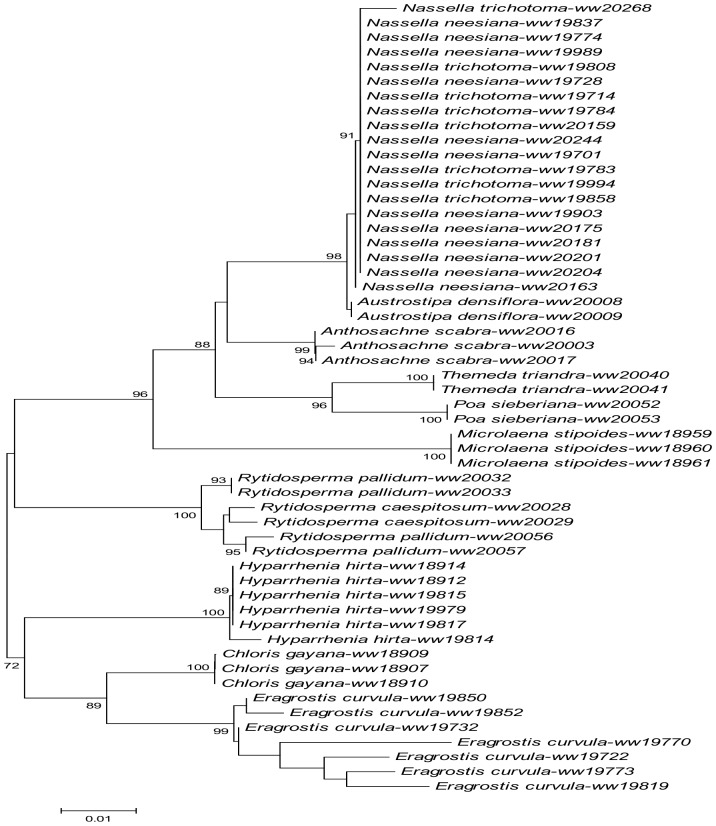
NJ trees inferred from six loci. **A: *atpF*, B: *psbE*, C: *matK*, D:** ETS**, E: *ndhK*, F:** ITS**. Bootstrap support > 70% (N = 1000 replications) for clusters as reported. Scale bars indicate proportion of differences under a K2P model.**

**Fig 3:**
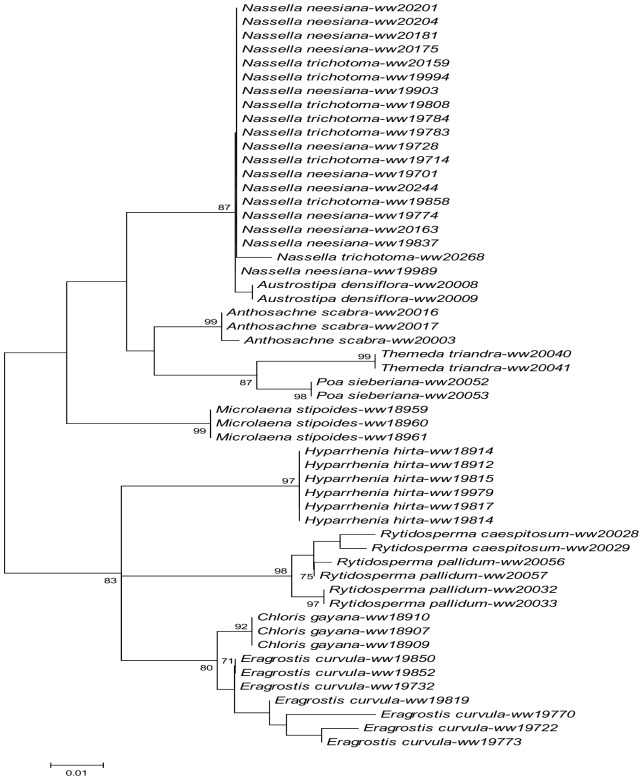
ML trees inferred from six loci. **A: *atpF*, B: *psbE*, C: *matK*, D:** ETS**, E: *ndhK*, F:** ITS**. Bootstrap supports > 70% (N = 1000 replications) for clades as reported. Scale bars report proportion of differences under a GTR model.**

For *psbE*, the monophyly of two invasive grasses, *H*. *hirta* and *E*. *curvula*, were well supported (95% and 99% bootstrap support) by the NJ analysis. Although the genus *Nassella* was supported as monophyletic, species within the genus (*N*. *neesiana* and *N*. *trichotoma*) were paraphyletic. All four invasive grasses which amplified at this gene were clearly distinguished from a single native species (*A*. *scabra*) (Fig 2B). Similar results were obtained from the ML tree of *psbE* (Fig 3B).

The higher PCR success rates in *atpF* (Table 2) made it possible to examine the distinguishing power of this locus across a wider range of grass species. Both NJ and ML trees of *atpF* supported the monophyly of three invasive species (*C*. *gayana*, *E*. *curvula*, *H*. *hirta*) and four native species (*M*. *stipoides*, *A*. *scabra*, *T*. *triandra*, *P*. *Sieberiana*), which means the majority of the examined taxa was distinguished by this locus. Although *atpF* failed to separate two invasive *Nasella* species (*N*. *neesiana* and *N*. *trichotoma*), it confirmed the monophyly of the *Nassella* genus and separated this genus from the native stipa species *A*. *densiflora* (Figs 2A and 3A). Similar results were obtained from the *ndhK* data (Figs 2E and 3E) but species coverage dropped slightly in the NJ and ML trees of *ndhK* (*C*. *gayana* and *A*. *scabra* were each represented by a single specimen, and *T*. *triandra* and *P*. *sieberiana* were not represented at all).

Similar to *atpF*, *matK* distinguished the invasive grasses of *H*. *hirta* and *E*. *curvula* from the tested native grasses (*A*. *scabra*, *P*. *sieberiana*, *T*. *triandra*, *M*. *stipoides*, *R*. *pallidum* and *R*. *caespitosum*). The monophyly of these native grasses were also confirmed except for the *T*. *triandra*, *R*. *pallidum and R*. *caespitosum* (Figs 2C and 3C). In addition, both NJ and ML trees of *matK* provided weak support to the monophyly of *N*. *trichotoma* and *N*. *neesiana*, and strong support for the separation of native stipa species (*A*. *densiflora*) from the two invasive *Nassella* species.

Two nuclear loci, ITS and ETS, were tested for their potential in distinguishing invasive grasses from native grasses in present study. While the PCR success rate for these two loci were relatively lower than that of the plasmid loci, the available sequence data from these two loci provided good distinguishing power in identifying different grass species. The NJ and ML trees of ITS clearly supported the monophyly of *E*. *curvula* and *C*. *gayana* whilst its counterparts of ETS provided strong support for the monophyly of *N*. *neesiana*, *N*. *trichotoma*, *C*. *gayana* and *H*. *hirta* (100% in both NJ and ML) (Figs 2D, 2F, 3D and 3F). The NJ and ML trees of ETS also separated the native stipa species (*A*. *densiflora*) from the invasive *Nassella* species with strong bootstrap support (100%).

### Multiple loci analysis

Four ETS related two loci combinations (ETS*—matK*, ETS*—ndhK*, ETS*–psbE* and ETS*–atpF*) strongly confirmed the monophyly of *N*. *neesiana* and *N*. *trichotoma*, which is an improvement relative to the results of single locus (*matK*, *ndhK*, *psbE* and *atpF*). On the contrary, *matK* related two loci combinations (except for *matK—psbE)* remain weak in distinguishing *N*. *neesiana* from *N*. *trichotoma*, although some combinations (*matK—atpF*, *matK—ndhK*, *matK—psbE*) were effective in confirming the monophyly of *E*. *curvula*, *H*. *hirta* and several native grass species. Monophyly of *E*. *curvula* was confirmed by all ITS related two loci combinations except for ETS—ITS (Table 5) (Trees not show).

**Table 5:** Overview of the multiple loci combinations analyses.

Combination of loci	Shared Specimens Number	Confirmed monophyletic species	Number of loci
ETS*—matK*	17	*N*. *trichotoma*, *N*. *neesiana*, *A*. *densiflora*	Di
ETS*—ndhK*	21	*N*. *trichotoma*, *N*. *neesiana*, *H*. *hirta*, *A*. *densiflora*	Di
ETS*—psbE*	17	*N*. *trichotoma*, *N*. *neesiana*, *H*. *hirta*	Di
ETS*—atpF*	25	*N*. *trichotoma*, *N*. *neesiana*, *H*. *hirta*, *A*. *densiflora*, *C*.*gayana*	Di
ITS*—matK*	15	*E*. *curvula*, *N*. *trichotoma*	Di
ITS*—ndhK*	18	*E*. *curvula*, *N*. *trichotoma*, *C*.*gayana*	Di
ITS*—psbE*	7	*E*. *curvula*, *N*. *trichotoma*	Di
ITS*—atpF*	11	*E*. *curvula*, *C*. *gayana*, *N*. *trichotoma*	Di
*matK—ndhK*	35	*E*. *curvula*, *H*. *hirta*, *M*. *stipoides*, *A*. *densiflora*	Di
*matK—psbE*	26	*N*. *neesiana*, *N*. *trichotoma*, *H*. *hirta*, *E*. *curvula*, *A*. *scabra*	Di
*matK—atpF*	34	*E*. *curvula*, *H*. *hirta*, *A*. *densiflora*, *A*. *scabra*, *P*. *sieberiana*, *M*. *stipoides*	Di
*ndhK—psbE*	27	*E*. *curvula*, *H*. *hirta*	Di
*ndhK—atpF*	35	*E*. *curvula*, *H*. *hirta*, *M*. *stipoides*, *C*. *gayana*, *A*. *scabra*	Di
*psbE—atpF*	26	*H*. *hirta*, *E*. *scabe*, *E*.*curvula*	Di
*atpF—ndhK—psbE*	22	*H*. *hirta*	Tri
ETS*—atpF—ndhK*	21	*N*. *trichotoma*, *N*. *neesiana*, *A*.*densiflora*, *H*. *hirta*	Tri
ETS*—matK—atpF*	17	*N*. *trichotoma*, *N*. *neesiana*, *A*.*densiflora*	Tri
ETS*—ndhK—psbE*	16	*N*. *trichotoma*, *N*. *neesiana*	Tri
*matK—atpF—ndhK*	24	*E*. *curvula*, *M*. *stipoides*, *A*. *densiflora*	Tri
*matK—ndhK—psbE*	22	*E*. *curvula*	Tri
*ndhK—*ITS*—matK*	14	*E*. *curvula*	Tri
*psbE—matK—atpF*	20	*A*. *scabra*, *H*. *hirta*	Tri
ETS*—matK—atpF—ndhK*	17	*N*. *trichotoma*, *N*. *neesiana*, *A*. *densiflora*	Tetra
ETS*—atpF—ndhK—psbE*	16	*N*. *trichotoma*, *N*. *neesiana*	Tetra
ETS*—matK—atpF—ndhK—psbE*	14	*N*. *trichotoma*, *N*. *neesiana*	Penda

Similar results were obtained from other combinations (tri, tetra and penta) of the six loci. Two *Nassella* invasive grasses, *N*. *neesiana* and *N*. *trichotoma*, were clearly distinguished by loci combinations consisting the ETS locus (three tri combinations: ETS*—atpF—ndhK*, ETS*—matK—atpF*, and ETS*—ndhK—psbE*; two tetra combinations: ETS*—matK—atpF—ndhK* and ETS*—atpF—ndhK—psbE*; and one penta combination: ETS*—matK—atpF—ndhK—psbE*). In addition, multiple loci combinations consisting of ITS failed to separate all grasses species except for *E*. *curvula* (Table 5) (Trees not show).

## Discussion

In present study, we tested six loci for their utility as DNA barcode targets to distinguish five invasive grass species from seven native grasses which frequently co-occur in eastern Australia. Among these, *matK* is recommended as one of two core loci by CBOL [14] for plant DNA barcoding. Our results (DNA barcode gap analysis, NJ and ML phylogenetic analyses) indicated that *matK* was suitable for distinguishing invasive *H*. *hirta* and *E*. *curvula* from native grasses (*M*. *stipoides*, *A*. *scabra*, *T*. *triandra*, *P*. *sieberiana*), but provided no or weak support for distinguishing *N*. *neesiana* from *N*. *trichotoma*, which are two important invasive grasses.

Similar results were obtained from *ndhK*, but the length of this locus (356 bp) is shorter than the recommended DNA barcode length [30]. The relatively lower PCR success rate of *ndhK* also limits its use as a general DNA barcode locus across the surveyed species. In contrast highest PCR success was achieved at *atpF*, and most of the examined taxa were distinguished (except for the two *Nassella* species) by this locus, indicating its potentials as a promising DNA barcode locus for the grasses of concern.

The remaining chloroplast locus, *psbE*, had longer sequence length (687 bp) and a high proportion (57.6%) of informative sites, but low PCR success rates across the examined species, which limits its utility as a general DNA barcode for grasses. As evidenced at other plastid loci, *psbE* provided no resolution in distinguishing *N*. *neesiana* from *N*. *trichotoma*.

Among the nuclear loci, ITS has been frequently reported as a potentially useful locus for plant DNA barcoding [31, 32], including its use for identification of stipoid grasses [4]. However, we experienced difficulties in amplifying this locus across multiple grass species. Our previous study [1] reported 75% fungal contamination rates among sequenced PCR products when using non-specific and universal ITS primers (ITS 5aF—ITS4R). Similarly fungal contamination with ITS primers has also been reported by Hollingsworth *et al*. [33]. However, in the present study, we have successfully eliminated the fungal contamination using the new primer set of ITS 26SE—ITS S3 together with modified PCR cycling conditions described previously[18]. Nevertheless, PCR and sequencing success rates using this new primer set remained low (average 20.5%), and the limited number of retrieved sequences failed to distinguish between *N*. *neesiana* from *N*. *trichotoma*, despite its success in distinguishing *E*. *curvula* and *C*. *gayana*.

The other nuclear locus, ETS, outperformed ITS in many ways, including the relatively higher PCR and sequencing success rate, and the power to distinguish three invasive grass species (*N*. *neesiana*, *N*. *trichotoma* and *H*. *hirta*). This locus could be a promising marker for grass DNA barcoding if more robust primers are designed for this locus to increase its PCR success rate (particularly for *E*. *curvula*).

Results of our PCR screening and DNA barcode gap analyses indicated no single locus can be universally used as a DNA barcode for distinguishing the grass species examined here. Loci examined either failed to amplify a portion of species or to resolve genetic limits among the species. Greater species resolution was in some cases obtained when combined loci analyses were employed. For example, *matK* confirmed the monophyly of multiple grass species except for *N*. *neesiana* and *N*. *trichotoma*, whilst ETS confirmed the monophyly of *N*. *neesiana* and *N*. *trichotoma* but failed to amplify *E*. *curvula*. When the DNA sequences of these two loci were concatenated and jointly analyzed, species monophyly of *N*. *trichotoma*, *N*. *neesiana* and *A*. *densiflora* were confirmed (Table 5). We noticed that the monophyly of *N*. *neesiana* and *N*. *trichotoma* were confirmed by all ETS related loci combinations (except for those combinations consisting both ETS and ITS), but were not confirmed by other loci combinations without the component of ETS. This suggests that ETS plays a crucial role in allowing identification of the two *Nassella* species in the multiple loci combinations, and could be a useful 2^nd^ locus in combined analyses to improve accuracy of invasive species identifications.

In summary, the present studies evaluated the distinguishing power of six loci (ETS, ITS, *atpF*, *matK*, *ndhK* and *psbE*) for DNA barcoding of five invasive weeds and seven native grasses, which co-occur in eastern Australia. Among the four plastid loci, *atpF* and *matK* showed higher PCR rates and better distinguishing power than the remaining loci, making them suitable for further consideration as promising DNA barcodes of the targeted grass species. Among the two nuclear loci, ETS showed better potential as a DNA barcode for the separation of two invasive *Nassella* species. We conclude that a dual locus DNA barcode combination of *atpF* and *matK* may be used to genetically distinguish several prominent invasive grass species present in eastern Australia from co-occurring native grasses often mistaken for the invasive types. Furthermore, use of the ETS locus as a DNA barcode for genetic separation and identification of the two *Nassella* spp. may provide some application in future screening of those two WONs species; arguably further optimization of this locus may also allow it to be used for DNA barcode assay and identification of a broader assemblage of native and invasive grass species in Australia.

## Supporting information

S1 Table Plant material, collection details and GenBank accession numbers of material used for comparative evaluation of six candidate DNA barcoding regions in five Invasive grasses and seven native grasses. All specimens are housed at WWAI unless otherwise indicated; N/A—indicates no sequence was obtained.
(DOCX)
