# Supplementary material for: Evaluation of six candidate DNA barcode loci for identification of five important invasive grasses in eastern Australia
Source: PLoS One. 2017 Apr 11;12(4):e0175338. doi: 10.1371/journal.pone.0175338 (PMC5388481; doi:10.1371/journal.pone.0175338)
Supplement: S1 Table — All specimens are housed at WWAI unless otherwise indicated; N/A—indicates no sequence was obtained. (DOCX) [file pone.0175338.s001.docx]

**S1 Table**

Plant material, collection details and GenBank accession numbers of material used for comparative evaluation of six candidate DNA barcoding regions in five Invasive grasses and seven native grasses. All specimens are housed at WWAI unless otherwise indicated; N/A – indicates no sequence was obtained

| **Species** | **Category** | **Source of materials** | **Location in Australia** | **Latitude** | **Longitude** | **Voucher** | **Collectors** | ***matK*** | **ITS** | **ETS** | ***atpF*** | ***ndhK*** | ***psbE*** |
| --- | --- | --- | --- | --- | --- | --- | --- | --- | --- | --- | --- | --- | --- |
| *Chloris gayana* | Invasive weeds | Fresh leave | Wagga Wagga, New South Wales | -35.103 | 147.3493 | ww18907 | Wang. A. & Crocker, J. | N/A | KX281082 | N/A | KY079318 | N/A | N/A |
| *Chloris gayana* | Invasive weeds | Fresh leave | Wagga Wagga, New South Wales | -35.103 | 147.3493 | ww18909 | Wang. A. & Crocker, J. | N/A | KX281080 | KX281103 | KY079313 | N/A | N/A |
| *Chloris gayana* | Invasive weeds | Fresh leave | Wagga Wagga, New South Wales | -35.103 | 147.3493 | ww18910 | Wang. A. & Crocker, J. | N/A | KX281066 | N/A | KY079288 | KX434534 | N/A |
| *Hyparrhenia hirta* | Invasive weeds | Fresh leave | Wagga Wagga, New South Wales | -35.124 | 147.309 | ww18912 | Wang, A. & Crocker, J. | KX290970 | N/A | N/A | KY079299 | N/A | KX360616 |
| *Hyparrhenia hirta* | Invasive weeds | Fresh leave | Wagga Wagga, New South Wales | -35.1238 | 147.3088 | ww18914 | Wang. A. & Crocker, J. | KX290963 | N/A | N/A | KY079293 | KX434539 | KX360613 |
| *Microlaena stipoides* | Native grass | Fresh leave | Cheltenham , Victoria | -37.9504 | 145.0595 | ww18959 | Chivers, I. | KX290987 | N/A | N/A | KY079315 | KX434560 | N/A |
| *Microlaena stipoides* | Native grass | Fresh leave | Cheltenham , Victoria | -37.9504 | 145.0595 | ww18960 | Chivers, I. | KX290988 | N/A | N/A | KY079316 | N/A | N/A |
| *Microlaena stipoides* | Native grass | Fresh leave | Cheltenham , Victoria | -37.9504 | 145.0595 | ww18961 | Chivers, I. | KX290979 | N/A | N/A | KY079306 | KX434552 | N/A |
| *Nassella neesiana* | Invasive weeds | Fresh leave | Walla Walla, New South Wales | -35.7928 | 146.9168 | ww19701 | Wang, A., Deane, P. & Hibberson, N. | KX290984 | N/A | N/A | KY079311 | KX434557 | KX360624 |
| *Nassella trichotoma* | Invasive weeds | Fresh leave | Bungowannah, New South Wales | -35.9396 | 146.7596 | ww19714 | Wang, A., Deane, P. & Hibberson, N. | KX290986 | KX281081 | KX281104 | KY079314 | KX434559 | KX360626 |
| *Eragrostis curvula* | Invasive weeds | Fresh leave | Table top, New South Wales | -35.9302 | 147.0023 | ww19718 | Wang, A., Deane, P. & Hibberson, N. | KX290969 | KX281074 | N/A | N/A | KX434545 | KX360615 |
| *Eragrostis curvula* | Invasive weeds | Fresh leave | Table top, New South Wales | -35.9302 | 147.0023 | ww19720 | Wang, A., Deane, P. & Hibberson, N. | KX290976 | KX281076 | N/A | N/A | KX434549 | KX360618 |
| *Eragrostis curvula* | Invasive weeds | Fresh leave | Corowa, New South Wales | -36.0341 | 146.3695 | ww19722 | Wang, A., Deane.P. & Minogue, P. | KX290966 | KX281071 | N/A | KY079297 | KX434543 | N/A |
| *Eragrostis curvula* | Invasive weeds | Fresh leave | Corowa, New South Wales | -36.0341 | 146.3695 | ww19723 | Wang, A., Deane.P. & Minogue, P. | KX290962 | KX281068 | N/A | N/A | KX434537 | N/A |
| *Eragrostis curvula* | Invasive weeds | Fresh leave | Corowa, New South Wales | -36.0341 | 146.3695 | ww19724 | Wang, A., Deane.P. & Minogue, P. | KX290967 | KX281073 | N/A | N/A | KX434544 | N/A |
| *Eragrostis curvula* | Invasive weeds | Fresh leave | Corowa, New South Wales | -36.0341 | 146.3695 | ww19725 | Wang, A., Deane.P. & Minogue, P. | KX290994 | KX281084 | N/A | N/A | KX434566 | KX360632 |
| *Nassella neesiana* | Invasive weeds | Fresh leave | Corowa, New South Wales | -35.9599 | 146.4118 | ww19728 | Wang, A., Deane.P. & Minogue, P. | KX290957 | N/A | KX281091 | KY079287 | KX434533 | KX360611 |
| *Eragrostis curvula* | Invasive weeds | Fresh leave | Cooma, New South Wales | -36.2403 | 149.1418 | ww19732 | Wang, A. & Jones, B. | KX290985 | KX281079 | N/A | KY079312 | KX434558 | KX360625 |
| *Eragrostis curvula* | Invasive weeds | Fresh leave | Canberra, Australian Capital Territory | -35.338 | 149.102 | ww19760 | Wang, A. & Talyer, S. | N/A | KX281072 | N/A | N/A | N/A | N/A |
| *Eragrostis curvula* | Invasive weeds | Fresh leave | Goubourn, New South Wales | -34.7597 | 149.7371 | ww19766 | Wang, A. & Ross, M. | KX290982 | KX281078 | N/A | N/A | KX434554 | KX360621 |
| *Eragrostis curvula* | Invasive weeds | Fresh leave | Goubourn, New South Wales | -34.7597 | 149.7371 | ww19767 | Wang, A. & Ross, M. | KX290978 | KX281077 | N/A | N/A | KX434551 | N/A |
| *Eragrostis curvula* | Invasive weeds | Fresh leave | Goubourn, New South Wales | -34.7597 | 149.7371 | ww19768 | Wang, A. & Ross, M. | KX290959 | KX281067 | N/A | N/A | KX434535 | N/A |
| *Eragrostis curvula* | Invasive weeds | Fresh leave | Goubourn, New South Wales | -34.7597 | 149.7371 | ww19769 | Wang, A. & Ross, M. | KX290965 | KX281070 | N/A | N/A | KX434541 | KX360614 |
| *Eragrostis curvula* | Invasive weeds | Fresh leave | Goubourn, New South Wales | -34.7567 | 149.7395 | ww19770 | Wang, A. & Ross, M. | KX290972 | KX281075 | N/A | KY079301 | N/A | N/A |
| *Eragrostis curvula* | Invasive weeds | Fresh leave | Goubourn, New South Wales | -34.7567 | 149.7395 | ww19771 | Wang, A. & Ross, M. | KX291000 | KX281086 | N/A | N/A | KX434570 | N/A |
| *Eragrostis curvula* | Invasive weeds | Fresh leave | Goubourn, New South Wales | -34.7567 | 149.7395 | ww19773 | Wang, A. & Ross, M. | KX290996 | KX281085 | N/A | KY079327 | KX434567 | N/A |
| *Nassella neesiana* | Invasive weeds | Fresh leave | Goubourn, New South Wales | -34.757 | 149.726 | ww19774 | Wang, A.& Ross, M. | KX290977 | N/A | KX281099 | KY079305 | KX434550 | KX360619 |
| *Nassella trichotoma* | Invasive weeds | Fresh leave | Goubourn, New South Wales | -34.7342 | 149.7173 | ww19783 | Wang, A. & Ross, M. | KX290973 | N/A | KX281096 | KY079302 | KX434546 | N/A |
| *Nassella trichotoma* | Invasive weeds | Fresh leave | Goubourn, New South Wales | -34.7342 | 149.7173 | ww19784 | Wang, A. & Ross, M. | KX290952 | N/A | KX281088 | KY079282 | KX434529 | KX360607 |
| *Nassella trichotoma* | Invasive weeds | Fresh leave | Camden, New South Wales | -34.0795 | 150.67 | ww19808 | Wang, A. & McNaughton, M. | N/A | N/A | KX281095 | KY079296 | KX434542 | N/A |
| *Hyparrhenia hirta* | Invasive weeds | Fresh leave | Picton, New South Wales | -34.1625 | 150.6184 | ww19814 | Wang, A. & Burgess-Buxton, A. | N/A | N/A | KX281094 | KY079295 | KX434540 | N/A |
| *Hyparrhenia hirta* | Invasive weeds | Fresh leave | Picton, New South Wales | -34.1625 | 150.6184 | ww19815 | Wang, A. & Burgess-Buxton, A. | N/A | N/A | N/A | KY079334 | KX434572 | KX360636 |
| *Hyparrhenia hirta* | Invasive weeds | Fresh leave | Picton, New South Wales | -34.1625 | 150.6184 | ww19817 | Wang, A. & Burgess-Buxton, A. | N/A | N/A | N/A | KY079335 | KX434573 | KX360637 |
| *Eragrostis curvula* | Invasive weeds | Fresh leave | Picton, New South Wales | -34.1473 | 150.6715 | ww19819 | Wang, A. & Burgess-Buxton, A. | N/A | KX281083 | N/A | KY079321 | KX434563 | N/A |
| *Nassella neesiana* | Invasive weeds | Fresh leave | Bungendore, New South Wales | -35.246 | 149.458 | ww19837 | Wang, A. & Plumb, N. | KX290955 | N/A | KX281089 | KY079285 | KX434531 | KX360609 |
| *Eragrostis curvula* | Invasive weeds | Fresh leave | Wagga Wagga, New South Wales | -35.0705 | 147.3795 | ww19850 | Wang. A. & Crocker, J. | N/A | KX281087 | N/A | KY079331 | KX434571 | N/A |
| *Eragrostis curvula* | Invasive weeds | Fresh leave | Wagga Wagga, New South Wales | -35.0705 | 147.3795 | ww19852 | Wang. A. & Crocker, J. | N/A | KX281069 | N/A | KY079291 | KX434538 | N/A |
| *Nassella trichotoma* | Invasive weeds | Fresh leave | Wagga Wagga, New South Wales | -34.978 | 147.4388 | ww19858 | Wang. A. & Crocker, J. | KX290956 | N/A | KX281090 | KY079286 | KX434532 | KX360610 |
| *Nassella neesiana* | Invasive weeds | Fresh leave | Armidale, New South Wales | -30.5023 | 151.6735 | ww19903 | Shephard, A. | KX290983 | N/A | KX281102 | KY079310 | KX434556 | KX360623 |
| *Hyparrhenia hirta* | Invasive weeds | herbarium specimens | Stanthorpe, Queensland | -28.7975 | 151.5583 | ww19979 | Batianoff, G.N. | N/A | N/A | KX281108 | KY079323 | KX434564 | KX360629 |
| *Nassella neesiana* | Invasive weeds | Fresh leave | Armidale, New South Wales | -30.517 | 151.5 | ww19989 | Telford I.R. | N/A | N/A | KX281107 | KY079322 | N/A | N/A |
| *Nassella trichotoma* | Invasive weeds | herbarium specimens | Throsbyn Capital Territory | -35.1681 | 149.1542 | ww19994 | Crawford, I. | KX290991 | N/A | KX281106 | KY079320 | KX434562 | KX360628 |
| *Anthosachne scabra* | Native grass | Fresh leave | Cheltenham , Victoria | -37.9504 | 145.0595 | ww20003 | Chivers, I. | KX290993 | N/A | N/A | KY079325 | N/A | KX360631 |
| *Austrostipa densiflora* | Native grass | Fresh leave | Wagga Wagga, New South Wales | -35.1364 | 147.3693 | ww20008 | Orchard, D. | KX290960 | N/A | KX281092 | KY079290 | KX434536 | N/A |
| *Austrostipa densiflora* | Native grass | Fresh leave | Wagga Wagga, New South Wales | -35.1364 | 147.3693 | ww20009 | Orchard, D. | KX290975 | N/A | KX281098 | KY079304 | KX434548 | N/A |
| *Anthosachne scabra* | Native grass | Fresh leave | Wagga Wagga, New South Wales | -35.1289 | 147.372 | ww20016 | Orchard, D. | KX291001 | N/A | N/A | KY079332 | N/A | KX360635 |
| *Anthosachne scabra* | Native grass | Fresh leave | Wagga Wagga, New South Wales | -35.1289 | 147.372 | ww20017 | Orchard, D. | KX290953 | N/A | N/A | KY079283 | KX434530 | KX360608 |
| *Rytidosperma caespitosum* | Native grass | Fresh leave | Tarcutta, New South Wales | -35.2477 | 147.6828 | ww20028 | Orchard, D. | KX290964 | N/A | N/A | KY079294 | N/A | N/A |
| *Rytidosperma caespitosum* | Native grass | Fresh leave | Tarcutta, New South Wales | -35.2477 | 147.6828 | ww20029 | Orchard, D. | KX290958 | N/A | N/A | KY079289 | N/A | N/A |
| *Rytidosperma pallidum* | Native grass | Fresh leave | Tarcutta, New South Wales | -35.2477 | 147.6828 | ww20032 | Orchard, D. | KX290954 | N/A | N/A | KY079284 | N/A | N/A |
| *Rytidosperma pallidum* | Native grass | Fresh leave | Tarcutta, New South Wales | -35.2477 | 147.6828 | ww20033 | Orchard, D. | KX291002 | N/A | N/A | KY079333 | N/A | N/A |
| *Themeda triandra* | Native grass | Fresh leave | Big Springs, New South Wales | -35.3397 | 147.3488 | ww20040 | Orchard, D. | KX290971 | N/A | N/A | KY079300 | N/A | N/A |
| *Themeda triandra* | Native grass | Fresh leave | Big Springs, New South Wales | -35.3397 | 147.3488 | ww20041 | Orchard, D. | KX290980 | N/A | N/A | KY079307 | N/A | N/A |
| *Poa sieberiana* | Native grass | Fresh leave | Kyeamba, New South Wales | -35.4862 | 147.6376 | ww20052 | Orchard, D. | KX290968 | N/A | N/A | KY079298 | N/A | N/A |
| *Poa sieberiana* | Native grass | Fresh leave | Kyeamba, New South Wales | -35.4862 | 147.6376 | ww20053 | Orchard, D. | KX290995 | N/A | N/A | KY079326 | N/A | N/A |
| *Rytidosperma pallidum* | Native grass | Fresh leave | Kyeamba, New South Wales | -35.4733 | 147.6369 | ww20056 | Orchard, D. | KX290997 | N/A | N/A | KY079328 | N/A | N/A |
| *Rytidosperma pallidum* | Native grass | Fresh leave | Kyeamba, New South Wales | -35.4733 | 147.6369 | ww20057 | Orchard, D. | KX290990 | N/A | N/A | KY079319 | N/A | N/A |
| *Anthosachne scabra* | Native grass | Fresh leave | Junee, New South Wales | -34.7286 | 147.6376 | ww20065 | Orchard, D. | KX290961 | N/A | N/A | N/A | N/A | N/A |
| *Nassella trichotoma* | Invasive weeds | Fresh leave | Galong, New South Wales | -34.7667 | 148.8577 | ww20159 | Deane, P. | KX290999 | N/A | KX281111 | KY079330 | KX434569 | KX360634 |
| *Nassella neesiana* | Invasive weeds | Fresh leave | Tamworth, New South Wales | -31.0881 | 150.9251 | ww20163 | Shephard, A. | KX290989 | N/A | KX281105 | KY079317 | KX434561 | KX360627 |
| *Nassella neesiana* | Invasive weeds | Fresh leave | Tamworth, New South Wales | -31.1701 | 150.0154 | ww20175 | Shephard, A. | KX290974 | N/A | KX281097 | KY079303 | KX434547 | KX360617 |
| *Nassella neesiana* | Invasive weeds | Fresh leave | Tamworth, New South Wales | -31.2032 | 151.0354 | ww20181 | Shephard, A. | KX290992 | N/A | KX281109 | KY079324 | KX434565 | KX360630 |
| *Nassella neesiana* | Invasive weeds | Fresh leave | Bathurst, New South Wales | -33.477 | 149.5934 | ww20201 | Deane, P. | N/A | N/A | KX281101 | KY079309 | KX434555 | KX360622 |
| *Nassella neesiana* | Invasive weeds | Fresh leave | Bathurst, New South Wales | -33.477 | 149.5934 | ww20204 | Deane, P. | KX290981 | N/A | KX281100 | KY079308 | KX434553 | KX360620 |
| *Nassella neesiana* | Invasive weeds | herbarium specimens | Tuganina, Victoria | -37.832 | 144.7642 | ww20244 | Costello, C. | KX290998 | N/A | KX281110 | KY079329 | KX434568 | KX360633 |
| *Nassella trichotoma* | Invasive weeds | herbarium specimens | Heathmont, Victoria | -37.8336 | 145.2442 | ww20268 | Reid, J.C. | N/A | N/A | KX281093 | KY079292 | N/A | KX360612 |
